# High-resolution genomic analysis: the tumor-immune interface comes into focus

**DOI:** 10.1186/s13059-015-0631-3

**Published:** 2015-03-31

**Authors:** Jonathan J Havel, Timothy A Chan

**Affiliations:** Human Oncology and Pathogenesis Program, Memorial Sloan Kettering Cancer Center, New York, NY 10065 USA; Department of Radiation Oncology, Memorial Sloan Kettering Cancer Center, New York, NY 10065 USA

## Abstract

A genomic analysis of heterogeneous colorectal tumor samples has uncovered interactions between immunophenotype and various aspects of tumor biology, with implications for informing the choice of immunotherapies for specific patients and guiding the design of personalized neoantigen-based vaccines.

Please see related article: http://dx.doi.org/10.1186/s13059-015-0620-6

Immunotherapy is a promising new approach for treating human malignancies. Approximately 20% of melanoma and lung cancer patients receiving immune checkpoint inhibitors show responses [1,2]. Current major challenges include identification of patients most likely to respond to specific therapies and elucidation of novel targets to treat those who do not. To address these problems, a detailed understanding of the dynamic interactions between tumors and the immune system is required. In a new study, Zlatko Trajanoski and colleagues [3] describe a powerful approach to dissecting these issues through high-resolution analysis of patient genomic data. This study represents a significant advance over previous work from this group, which defined 28 immune-cell-type gene expression signatures and identified specific cell types as prognostic indicators in colorectal cancer (CRC) patients [4]. Here, the authors [3] integrate genomic analyses of CRC tumor molecular phenotypes, predicted antigenicity (called the ‘antigenome’), and immune-cell infiltration derived from multiple independent cohorts to gain refined insights into tumor-immune system interactions.

## Not all tumor-infiltrating lymphocytes are created equal

Past studies have used immune-staining techniques to determine associations between a limited set of infiltrating immune cells and patient survival [5] or tumor molecular phenotype [6]. Here, the authors [3] use gene set enrichment analysis (GSEA) of immune cell expression signatures to ascertain associations of 28 immune-cell populations with patient survival and tumor molecular phenotypes. Effector memory CD8^+^ and CD4^+^ T cells, natural killer cells, and activated dendritic cells are significantly associated with improved overall survival. Interestingly, although the authors’ previous work found no significant prognostic value of regulatory T cells (Tregs) or myeloid-derived suppressor cells (MDSCs) [4], negative associations of these cell types with overall survival are among the strongest relationships observed in the current study. It is possible that variations in sample collection and preparation may have contributed to this discrepancy. The conclusions, supported by the numerous animal studies demonstrating the importance of cell-mediated immunosuppression, are substantially strengthened by a much larger cohort size used in this study.

Another important observation is the association of specific immune cell subsets with CRC tumor stage and molecular phenotypes as classified by mutation rate, microsatellite instability, and methylation status. This knowledge will be crucial in determining which types of immunotherapy are most likely to benefit individual patients. Interestingly, although hypermutated microsatellite-unstable tumors show strong enrichment of adaptive immune cells, similar enrichment is notably lacking in the small population of hypermutated microsatellite-stable tumors. This raises an intriguing question of whether and how microsatellite instability/mismatch repair may independently shape immune responses. Furthermore, Trajanoski and colleagues [3] find that tumor-infiltrating lymphocytes transition from an adaptive to an innate immunophenotype with increasing tumor stage. This raises an interesting issue of whether immunotherapies that depend on the adaptive immune response can be effective in later stage CRC tumors.

## Diversity of tumor antigens

In addition to characterizing immune components involved in tumor immune responses, it is equally important to identify and understand the tumor-associated antigens that elicit these responses, called the ‘antigenome’. The authors [3] analyze RNA-seq and genomic data to identify two types of tumor antigens in CRC - non-mutated cancer germline antigens that are aberrantly overexpressed, and neoantigens, which are generated from non-synonymous somatic mutations. Importantly, the authors [3] find that cancer-germline antigens are highly shared among patients and are independent of molecular and immune phenotype. In contrast, neoantigens are enriched in the hypermutated microsatellite-unstable phenotype tumors and rarely shared among patients. These results imply a heightened importance of neoantigens in comparison to cancer-germline antigens [7]. In addition, similar analytical methods have recently been applied to identify functional neoantigens in human melanoma and cholangiocarcinoma [8-10]. An emerging theme of these studies is that the *in vitro* validation rate for predicted neoantigens is relatively low; however, it is unclear whether this is due to limited sensitivity of functional assays or epigenetic silencing to circumvent immunoediting, or whether the number of immunogenic neoantigens is in fact small. Interestingly, Trajanoski and colleagues [3] find a modest yet significant decrease in neoantigen frequency with increasing tumor stage. Considering the concomitant decrease in adaptive immune cell infiltration, it is tempting to speculate that this phenomenon reflects immunoediting of critical neoepitopes during tumor progression. Furthermore, the authors find an association, albeit not statistically significant, between increased neoantigen burden and improved patient survival. This finding complements a recent report [9] showing that whereas neoantigen burden roughly predicts survival of anti-CTLA-4-treated melanoma patients, a collection of consensus neoepitope motifs is strongly associated with patient survival. It will be interesting to see if future studies can determine the effect of CRC neoantigen burden in the setting of immunotherapy, and answer the questions of whether an analogous signature of prognostic neoepitope motifs exists for CRC, and whether there are any similarities between substring signatures of different tumor types.

## Tumor escape mechanisms and new targets - one size does not fit all

All tumors evade immune system destruction through any of numerous possible mechanisms. Knowing which escape mechanism(s) to target in specific tumors is critically important for successful immunotherapy. The authors [3] analyze immune infiltrate composition and immune modulatory molecule expression in relation to molecular CRC tumor phenotypes. Importantly, they find that in hypermutated tumors, immunoinhibitory molecules such as CTLA-4 and PD-1 are upregulated, whereas immunosuppressive cells such as Tregs and MDSCs are depleted. This finding confirms previous studies demonstrating that increased mutational burden is associated with response to anti-CTLA-4 and anti-PD-1 therapies [9]. On the other hand, non-hypermutated tumors show increased immunosuppressive cell infiltration and downregulation of immunoinhibitory molecules [3], highlighting the need to develop Treg- and MDSC-directed therapies. Finally, the authors use two modeling techniques to identify BCMA (TNFRSF17) and CCR8 as putative novel targets for immunotherapy. CCR8 is identified as a determinant of tumor immunogenicity, and both molecules are positively associated with patient survival. It remains an intriguing question whether agonists of these molecules can improve response to immunotherapies.

## Conclusions

**Figure 1:**
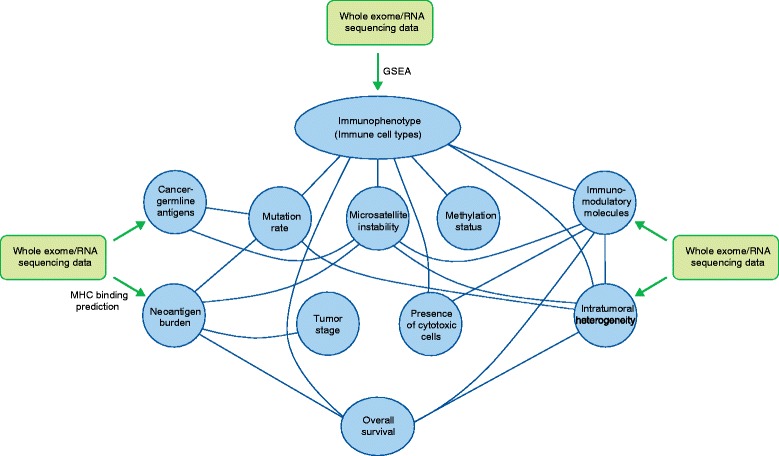
**Trajanoski and colleagues** **[** 3 **]** **use high-resolution genomic analysis to identify tumor immunophenotypes and predict tumor antigens.** Associations between these parameters and various molecular and clinical tumor phenotypes were determined, and are shown above as connecting lines. The findings corroborate previous work, generate new hypotheses regarding tumor-immune interactions, and identify putative novel targets for immunotherapy. GSEA, gene set enrichment analysis; MHC, major histocompatibility complex.

Trajanoski and colleagues [3] describe a fascinating new application of genomic analysis in heterogeneous tumor samples to uncover interactions among the immunophenotype and various aspects of CRC tumor biology. Extension of these analyses will help inform the choice of immunotherapies for specific patients and guide the design of personalized neoantigen-based vaccines. Although some of the authors’ findings have been demonstrated previously by other methods, the fact that all conclusions here are derived from computational analysis demonstrates the power and efficiency of such an approach (Figure 1). It will be of great value to apply these methods to datasets from other tumor types and in the context of response to immunotherapies. By using innovative genomic analyses, Trajanoski and colleagues [3] have shed new light on the clandestine world of immune cell-tumor interactions and bring us a step closer toward implementation of personalized cancer immunotherapies.

## Abbreviations

CRC: Colorectal cancer
CTLA-4: Cytotoxic T-lymphocyte-associated protein 4
GSEA: Gene set enrichment analysis
MDSCs: Myeloid-derived suppressor cells
PD-1: Programmed cell death protein 1
Treg: Regulatory T cell
